# Extreme Oncoplasty: Avoiding Mastectomy and Expanding the Limits of Breast Conservation—A Comparative Study of 718 Patients

**DOI:** 10.1245/s10434-026-20139-9

**Published:** 2026-07-13

**Authors:** Jenna Capuano, Melvin J. Silverstein, Nirav Savalia, Sadia Khan, Elizabeth Kraft

**Affiliations:** 1 Hoag Memorial Hospital Presbyterian, Newport Beach, CA USA; 2 Keck School of Medicine, University of Southern California, Los Angeles, CA USA

## Abstract

**Background:**

For many years, large, multifocal/multicentric tumors have routinely been treated with mastectomy. Extreme oncoplastic breast-conserving surgery attempts to achieve complete oncologic resection of large tumors while preserving the natural shape, symmetry, and function of breasts. This paper compares recurrence and survival probabilities for extreme and standard (≤50 mm) tumors.

**Methods:**

A prospective oncoplastic database accrued between 2008 and 2025 was queried for patients with breast cancer treated with reduction mammoplasty and whole breast irradiation. In total, 340 patients with extreme tumors >50 mm were compared with 378 patients with standard tumors ≤50 mm. Demographic and biologic factors and recurrence and survival probabilities were compared.

**Results:**

Biologic factors were statistically similar for both groups. Negative margins, defined as no ink on tumor, were achieved in 96.8% of standard and 92.6% of extreme cases (*p* = 0.01). Mean tumor span was 26.3 mm for standard and 74.3 mm for extreme cases (*p* < 0.001). Re-excision rates were 5.6 and 10.9%, for standard and extreme cases, respectively (*p* = 0.01). The 5-year local recurrence probability was 1.44% for standard cases versus 3.37% for extreme cases (*p* = 0.067). Distant recurrence, breast cancer-specific survival, and overall survival did not differ significantly.

**Conclusions:**

Extreme oncoplasty followed by whole breast irradiation allows safe breast conservation in selected patients with large, >50 mm, multifocal/multicentric tumors, achieving local and distant recurrence and survival outcomes comparable to those of standard oncoplastic cases. When technically feasible, breast conservation with extreme oncoplastic reconstruction should be considered a valid alternative to mastectomy in appropriately selected patients.

## Background

Breast-conserving therapy (BCT) combines breast-conserving surgery and adjuvant radiation therapy and yields survival outcomes equivalent to those with mastectomy for appropriately selected patients with breast cancer. Some contemporary studies suggest a modest survival advantage for patients receiving breast conservation with radiation therapy.^1^^,^^2^ Oncoplastic breast surgery, the combination of surgical excision with immediate plastic surgical reconstruction, achieves wider surgical margins and preserves breast shape, symmetry, and function.

Traditionally, tumors >50 mm, particularly when multifocal or multicentric, have been considered relative or absolute contraindications to breast conservation because of the potential for higher local recurrence and the difficulty obtaining adequate margin width. As a result, many of these patients are recommended for mastectomy.

Extreme oncoplasty, a term coined by Silverstein et al.,^3–5^ expands the application of BCT by incorporating advanced reconstructive techniques, allowing the resection of larger and more complex, >50 mm, multifocal/multicentric lesions that would otherwise require mastectomy. Despite the increasing adoption of extreme oncoplasty, published data are limited, with most studies consisting of small series, heterogeneous treatment approaches, inconsistent definitions, and relatively short follow-up.

This study compares long-term recurrence and survival probabilities between two groups of patients: those with extreme tumors, defined as breast cancer >50 mm that is often multicentric and/or multifocal versus standard tumors ≤50 mm. All patients were treated with oncoplastic breast-conserving reduction surgery and adjuvant whole breast radiation therapy (WBRT). To our knowledge, this is the largest series of extreme oncoplastic cases published to date.

## Methods

**Fig. 1:**
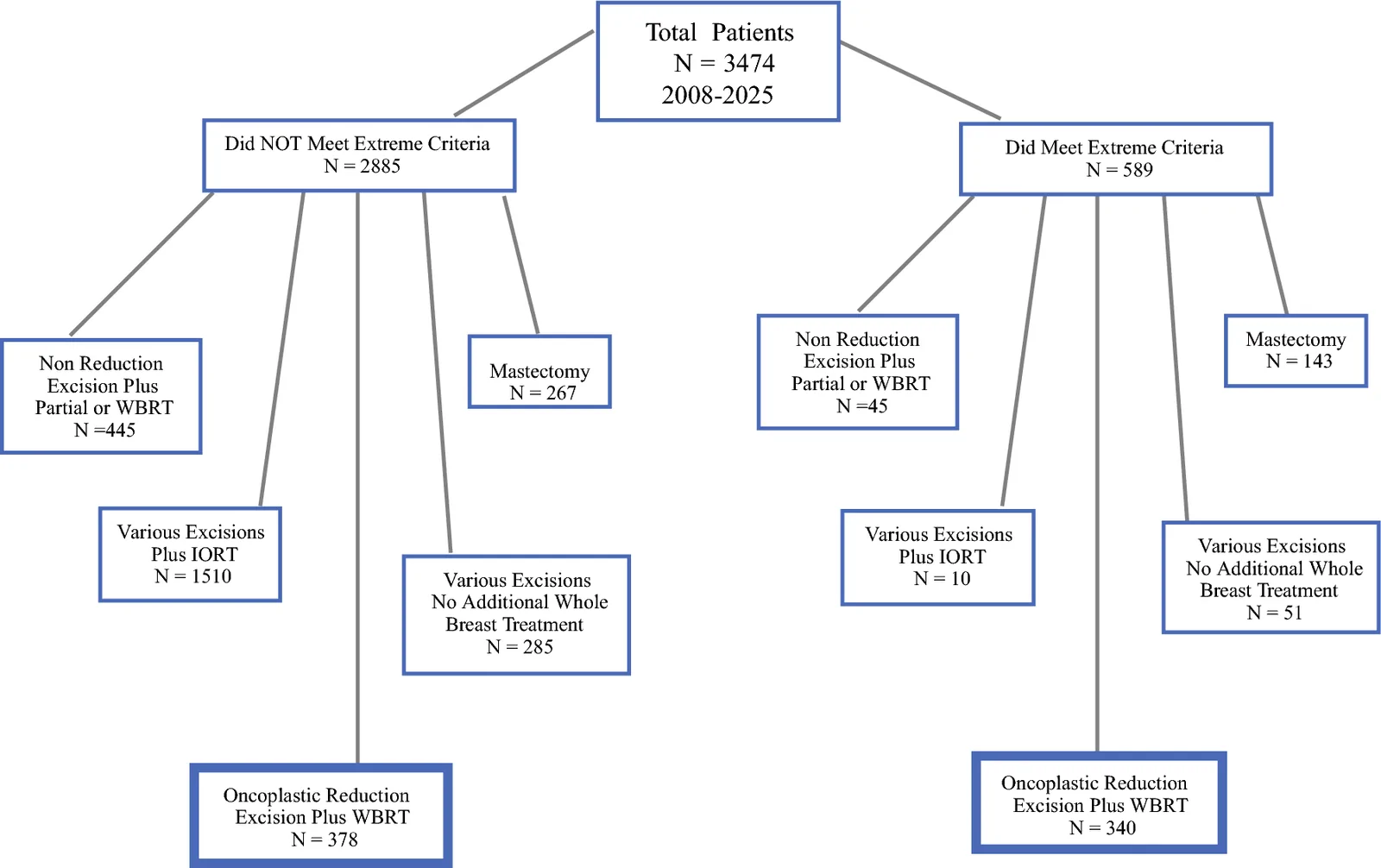
Flowchart of 3474 patients with breast cancer treated between 2008 and 2025. Patients are divided into standard cases (tumors ≤ 50 mm) and extreme cases (tumors > 50 mm). To be included in this study (indicated by the bottom two boxes outlined in bold), all patients had their tumors removed with a reduction excision and received postoperative whole breast radiation therapy

A prospective institutional review board–approved breast cancer database of 3474 patients treated between 2008 and 2025 was used to identify 718 patients with ductal carcinoma in situ, invasive ductal carcinoma, invasive lobular carcinoma, or mixed histologies who underwent oncoplastic excision using various forms of reduction mammoplasty followed by WBRT. We excluded patients who went directly to mastectomy or were converted to mastectomy, patients with non-oncoplastic lumpectomies, patients treated with intraoperative radiation therapy or partial breast irradiation, and patients who declined adjuvant WBRT (Fig. 1). The remaining 718 patients were stratified into two groups based on final tumor span: standard tumors ≤50 mm in total span and extreme tumors >50 mm in total span, often with multifocal or multicentric disease.

Final tumor span, or the maximum linear extent of disease, was determined by surgical pathology. For a single contiguous focus of tumor, tumor span was determined by direct pathologic measurement. For multifocal/multicentric disease, tumor span was calculated as the distance from the outside edge of the two most widely separated tumor foci. The tumor span was reported as “estimated tumor span” on the pathology report, and that number was used to divide the patients into standard and extreme categories. Non-contiguous lesions that spanned more than 50 mm by imaging and/or pathologic measurement were defined as extreme, even though the largest single focus might be smaller than 50 mm. For patients who received neoadjuvant chemotherapy and achieved a partial or complete response, the pre-treatment clinical size estimate, determined by imaging, was used to assign the patients into standard or extreme categories.

All patients underwent a standardized preoperative evaluation with digital mammography, breast ultrasound, and breast magnetic resonance imaging, followed by multidisciplinary review. The clinical size estimate was defined by the largest dimension using these imaging modalities. Preoperative surgical planning integrated clinical size, breast anatomy, and tumor biology.

Oncologic breast surgeons performed all tumor resections and axillary staging, if indicated according to contemporary oncologic guidelines. Plastic surgeons performed all oncoplastic reconstructions. All patients were treated with various forms of oncoplastic reduction mammoplasty.

Margin width was defined as the distance between tumor and ink. Final margin status reflected the final margin assessment from the index operation, including any additional shave margins excised at the time of initial surgery. No ink on tumor was considered acceptable for all subtypes, including ductal carcinoma in situ, since all patients went on to receive WBRT.

Adjuvant WBRT was given in all cases and was mandatory for inclusion. Systemic therapy (chemotherapy, hormone therapy, or targeted agents) was administered at the discretion of the patient’s medical oncologist based on tumor biology and contemporary guidelines.

The primary endpoints were local recurrence, distant recurrence, breast cancer-specific survival, and overall survival. Time-to-event outcomes were calculated from the date of diagnosis to date of last follow-up or event. Probabilities were estimated using the Kaplan–Meier method. Comparisons between curves were done using the log-rank test. Continuous variables were compared using Student’s *t*-test and categorical variables using the chi-squared test, with a two-sided p value < 0.05 considered statistically significant.

## Results

A total of 718 patients underwent oncoplastic breast-conserving surgery, of which 378 had standard tumors and 340 had extreme tumors; all received and completed adjuvant WBRT. Demographics and clinicopathologic variables are compiled in Table 1. Patients with extreme tumors were slightly younger (58.0 vs 60.4 years, *p* = 0.003), with similar rates of invasive disease (86.5 vs 89.7%, *p* = 0.18). The number of patients with invasive disease receiving neoadjuvant chemotherapy did not differ between the groups (27.1% for standard tumor vs 33.7% for extreme, *p* = 0.074). Tumor biology, including histologic subtype, tumor grade, receptor status, molecular subtype, the presence of lymphovascular invasion, and nodal status, are shown in Table 2. Although most of the biological factors were well balanced between the groups, a significantly higher percentage of patients with triple-negative breast cancer were identified in the standard group (12.4 vs 4.4% *p* <0.001). There was no statistical difference between groups for any of the other biologic factors in Table 2.

**Table 1:** Characteristics of trial cohorts

Characteristics	Standard	Extreme	*p*-Value
Total patients	378	340	
Average age, years	60.4	58.0	0.003
Invasive	339 (89.7)	294 (86.5)	0.18
Invasive ductal	299 (79.1)	241 (70.1)	0.01
Invasive lobular	40 (10.6)	53 (15.6)	0.05
DCIS	39 (10.3)	46 (13.5)	0.18
Median follow-up, years (Average)	7.76 (7.63)	7.36 (7.09)	0.038
No ink on tumor	366 (96.8)	315 (92.6)	0.011
Margin ≥1 mm	333 (88.1)	249 (73.2)	<0.001
Margin ≥2 mm	311 (82.3)	228 (67.1)	<0.001
Margin ≥2 mm, DCIS only	29/39 (74.4)	24/46 (52.2)	0.036
Re-excision	21 (5.6)	37 (10.9)	0.009
Local recurrences	12	19	0.15
Invasive local recurrences	10	12	0.49
Same quadrant local recurrences	8	17	0.04
Average largest dimension of excision specimen, mm	92	117	<0.001
Average pathological tumor span, mm^a^	26.3	74.3	<0.001
Weight, g	125	185	<0.001
Neoadjuvant chemotherapy, invasive only	92/339 (27.1)	99/294 (33.7)	0.074

Data are presented as *n* or *n* (%) unless otherwise indicated

^a^For patients who experienced a partial or complete response to chemotherapy, the largest pre-treatment imaging tumor size estimate was used to determine tumor span and classify patients into the standard or extreme group

DCIS, ductal carcinoma in situ

**Table 2:** Tumor biology

	Standard	Extreme	*p*-Value
Average nuclear grade	2.31	2.27	0.39
Average SBR score, invasive only	6.71	6.57	0.92
Average Nottingham Grade, invasive only	2.14	2.06	0.65
ER+	311 (82.3)	291 (85.6)	0.19
PR+	269 (71.2)	242 (71.2)	0.93
HER2+, invasive only	58/339 (17.1)	57/294 (19.4)	0.46
Triple negative, invasive only	42/339 (12.4)	13/294 (4.4)	<0.001
Luminal A, invasive only	163/339 (48.1)	138/294 (46.9)	0.71
LVI, invasive only	33/339 (9.7)	29/294 (9.9)	0.95
Node positive, invasive only^a^	112/331 (33.8)	101/290 (34.8)	0.80

Data are presented as *n* (%) unless otherwise indicated

ER+, estrogen receptor positive; HER2, human epidermal growth factor receptor 2; LVI, lymphovascular invasion; PR+, progesterone receptor positive; SBR, Scarff-Bloom-Richardson

^a^In 12 patients, no nodes were removed

Tumor burden, as estimated by pathologic span, differed significantly between cohorts, with greater mean tumor span (74.3 vs 26.3 mm, *p* < 0.001) and heavier specimen weight (185 vs 125 gm, *p* < 0.001) in the extreme group.

Negative margins, defined as no ink on tumor, were achieved in 96.8% of standard cases and 92.6% of extreme cases (*p* = 0.011), whereas wider margins ≥1 mm were obtained in 88.1% and 73.2% of cases, respectively (*p* < 0.001). The margin data listed in Table 1 represent the final margin status achieved during the index operation, including excision of any additional shave margins. Re-excision rates due to positive or close margins were higher in the extreme cohort (10.9 vs 5.6%, *p* = 0.009). Median follow-up was similar between groups (7.76 vs 7.36 years, *p* = 0.038).

**Fig. 2:**
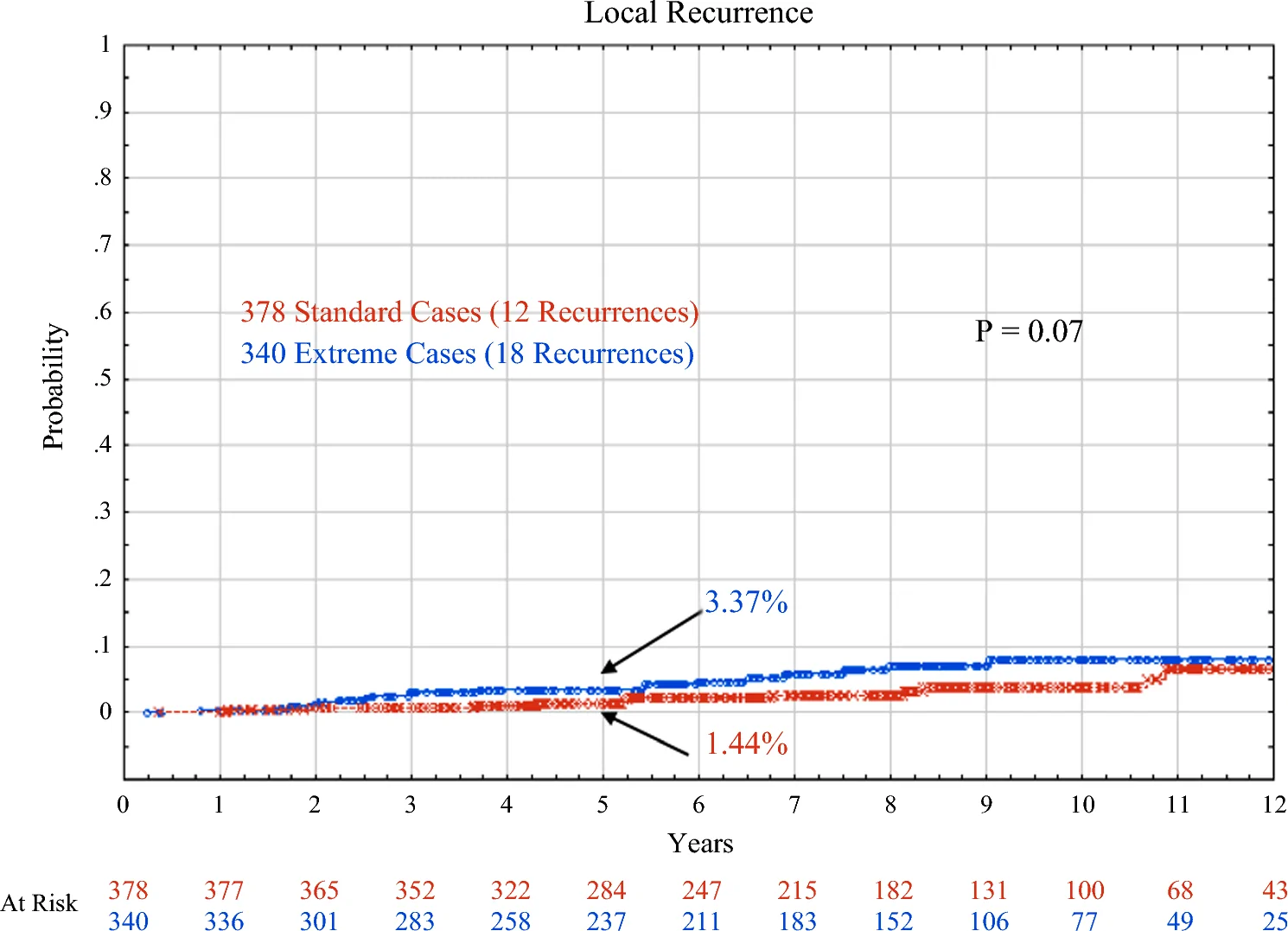
Kaplan-Meier probability of local recurrence for standard cases (tumors ≤ 50 mm) versus extreme cases (tumors > 50 mm). All patients had reduction excision of their tumors and received whole breast radiation therapy

**Fig. 3:**
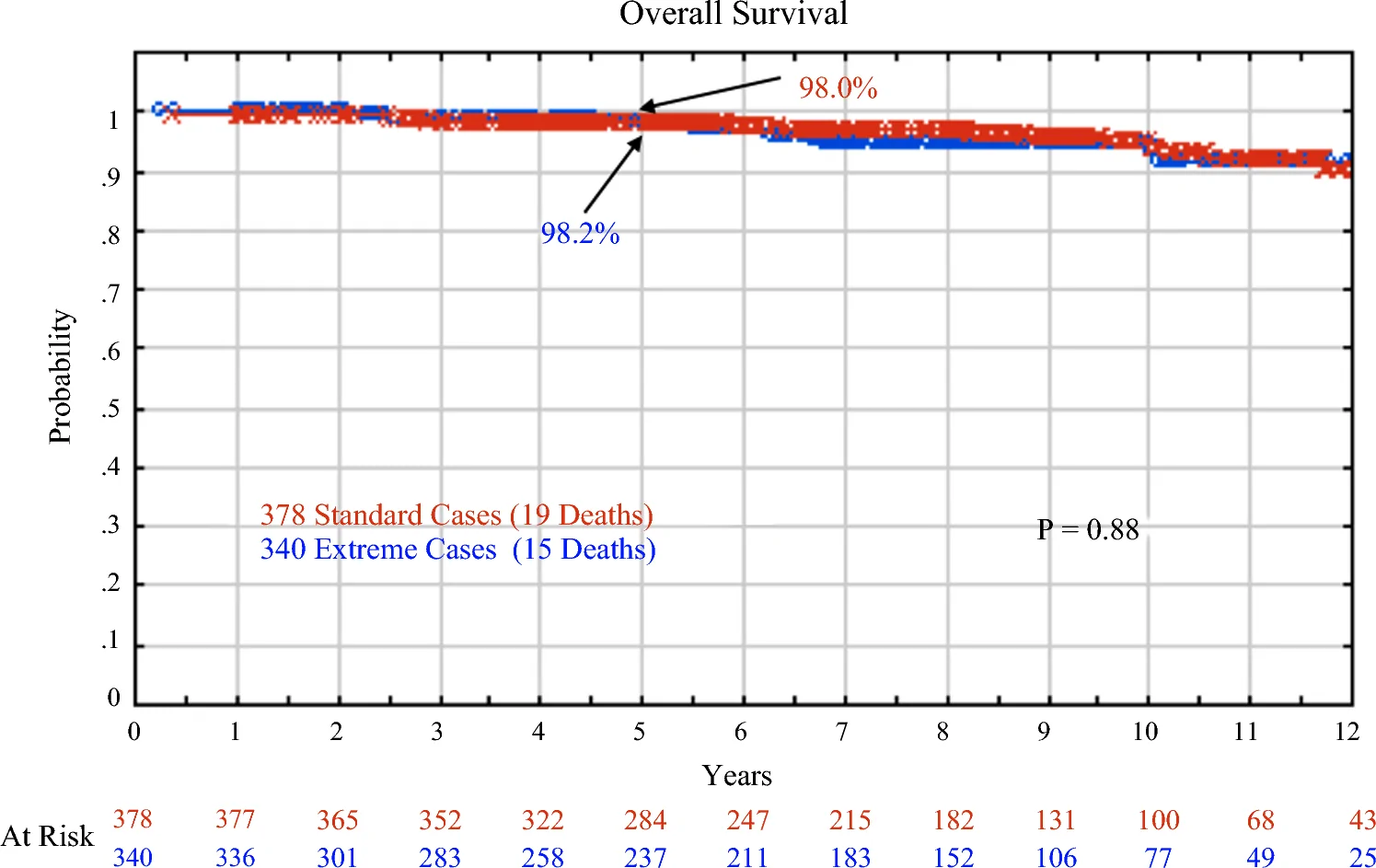
Kaplan-Meier probability of overall survival for standard cases (tumors ≤ 50 mm) versus extreme cases (tumors > 50 mm). All patients had reduction excision of their tumors and received whole breast radiation therapy

At 5 years, the Kaplan–Meier probability of local recurrence was 1.44% in the standard group and 3.37% in the extreme group (*p* = 0.067) (Fig. 2, Table 3). Distant recurrence probabilities were similar (5 years: 2.04% for the standard group vs 1.86% for the extreme group; *p* = 0.92). Overall survival did not differ between groups (5 years: 98.0% for the standard group vs 98.2% for the extreme group; *p* = 0.88), nor did breast cancer-specific survival (5 years: 98.6% for the standard group vs 98.2% for the extreme group; *p* = 0.96) (Fig. 3, Table 3).

**Table 3:** 5-year probability of recurrence and survival

5-Year probability	Standard	Extreme	*p*-Value
Local recurrence	1.44	3.37	0.067
Distant recurrence	2.04	1.86	0.92
Overall survival	98.0	98.2	0.88
Breast cancer-specific survival	98.6	98.2	0.96

Data are presented as percentages unless otherwise indicated

No differences were observed in invasive local recurrence, although same-quadrant recurrence was more frequent in the extreme cohort (*p* = 0.04). Despite the significantly greater tumor burden in the extreme group, the probabilities of distant recurrence, overall survival, and breast cancer-specific survival were statistically similar between groups.

## Discussion

Following the acceptance of breast conservation as an oncologically equal alternative to mastectomy, breast conservation was initially reserved for smaller, more favorable tumors.^6–9^ Over time, the acceptable tumor span for BCT increased. In 2015, Dr. Melvin Silverstein delivered the 50th IS Ravdin lecture at the American College of Surgeons Annual meeting^3^^,^^4^ and introduced the concept of extreme oncoplasty. Since that time, there has been a growing acceptance of this surgical approach.

Despite growing clinical acceptance, published literature validating the safety of extreme oncoplastic breast-conserving surgery remains limited. Existing data consist primarily of small, single-institution retrospective series with heterogeneous definitions, a lack of comparative cohorts, and short follow-up, thereby limiting conclusions regarding long-term oncologic safety.^10^^,^^11^

To date, only a few small series have evaluated the long-term safety of extreme oncoplasty. Pearce et al.^12^ evaluated 88 patients with extreme tumors over a median follow-up of 6.7 years. Although they demonstrated an encouraging 5-year local recurrence rate of 1.1%, the local recurrence rate rose notably to 16% at 10 years, and overall survival data were not reported.^12^ Similarly, Crown et al.^10^ reported on 111 patients who underwent extreme oncoplasty and received adjuvant radiation therapy, noting a local recurrence rate of 1.1% though they had short follow-up. In a comprehensive review by Vieira et al.,^13^ pooled data across available extreme oncoplasty literature demonstrated a recurrence rate <3.5%; however, among the subset of patients with >5 years of follow-up, the local recurrence rate escalated to 6%–9%. Survival data were not reported.^13^ Savioli et al.^11^ reported the outcomes of 50 patients who underwent extreme oncoplasty with a median follow-up of 62 months, reporting an overall recurrence rate of 10% (*n*=5). Interestingly, all five patients developed distant metastases, with only one patient also experiencing a concurrent local recurrence. All recurrences occurred in patients with large solitary tumors, >50 mm, rather than those with multifocal disease, and they reported a 95.7% breast cancer-specific survival rate.^11^ These studies highlight a paucity of outside institutional data regarding overall survival in extreme oncoplasty. The single-center study by Shaikh et al.^14^ represents one of the few exceptions and evaluated a high-risk cohort of 47 patients (40% node-positive and 25% triple-negative breast cancer) reporting a 5-year overall survival rate of 78%.

Our current study addresses several gaps in the literature. To our knowledge, this study is the largest series of extreme oncoplasty cases to date (*n* = 340) and directly compares extreme oncoplastic resections with standard oncoplastic resections (*n* =378) treated during the same time period, all of whom received WBRT. With a median follow-up of 7.5 years, we report outcome data on numerous oncologic endpoints, including local recurrence, distant recurrence, overall survival, and breast cancer-specific survival.

## Pathologic Outcomes and Margin Significance

Extreme cases were larger, spanning 74.3 mm vs 26.3 mm with a greater tumor burden (*p* <0.001). Because of this, they experienced a higher rate of close or involved margins and were re-excised more frequently. Nevertheless, distant recurrence, breast cancer-specific survival, and overall survival were statistically the same in both groups. Rates of negative margins, defined as no ink on tumor, exceeded 96% in standard cases and 92% in extreme cases (*p* = 0.011); although statistically significantly different, the lower rate of margin negativity for extreme cases did not translate into worse survival outcomes.

## Long-Term Oncologic Safety

Although local recurrence rates were marginally higher in extreme cases, the difference did not reach statistical significance, despite a trend toward significance (*p* = 0.067). With longer follow-up, it is likely that the higher local recurrence probability for extreme cases will become statistically significant. This should be expected because of their larger size, higher tumor burden, and higher frequency of close or involved surgical margins. Despite this, there was no impact on regional recurrence, distant recurrence, overall survival, or breast cancer-specific survival (all p-values not significant).

## The Case for Breast Conservation

Mastectomy should not be reflexively recommended for patients with large (>50 mm) or multifocal/multicentric tumors. The results reported by Hossino et al.^15^ suggest no survival disadvantage for extreme oncoplastic reduction surgery compared with mastectomy. Surgical management should instead be individualized in a multidisciplinary setting taking into account tumor biology, size and shape of the breast and tumor, position of the tumor, the feasibility of complete surgical resection, the planned reconstructive approach, the adequacy of residual breast tissue, the likelihood of achieving an acceptable oncologic and aesthetic outcome, and the patient’s preferences. Numerous papers suggest increased patient satisfaction and feelings of well-being with breast conservation using oncoplastic reduction compared with mastectomy.^16^

An added advantage supporting breast conservation in patients with large tumors is the potential to avoid post-mastectomy radiation therapy (PMRT) and its associated reconstructive morbidity. Patients with large tumors >50 mm and/or multicentric disease who undergo mastectomy frequently receive PMRT, particularly in the presence of nodal involvement or other high-risk features.

When implant-based reconstruction has been used, PMRT has been consistently associated with increased rates of complications, including capsular contracture, infection, reconstruction failure, and inferior cosmetic outcomes.^17^ PMRT increases the risk of fat necrosis, progressive tissue fibrosis, and symmetry-altering volume loss. Reported rates of capsular contracture after PMRT range from 20 to 50%.^18^ The rate of implant loss is significantly higher in patients receiving PMRT than in those receiving non-irradiated reconstructions. These complications often necessitate revisional surgery and may compromise long-term reconstructive outcomes. In contrast, successful breast conservation with postoperative radiation therapy avoids irradiation of an implant and decreases these risks.^19^^,^^20^ Alternatively, these risks can be mitigated with autologous reconstruction, as is often done. However, autologous reconstruction is far more resource intensive and often requires additional surgeries; the net result is an insensate breast with all the combined morbidity of mastectomy and autologous reconstruction. Extreme oncoplastic techniques offer the potential to preserve oncologic safety, with clinically meaningful improvements in psychosocial well-being and sexual well-being, reduced operative time, reduced PMRT-related complications, and minimized post-mastectomy pain syndrome, in a single outpatient surgery.^21^

Extreme oncoplastic surgery offers significant reconstructive advantages for patients who, despite extensive resection, are ultimately ineligible for breast conservation because of persistently positive margins. In these cases, the initial oncoplastic procedure reduces the redundant skin envelope and corrects ptosis, facilitating a safer and more aesthetic delayed conversion to a nipple-sparing mastectomy that might have otherwise been technically contraindicated.

This study has several limitations. As a single-institution analysis, the results may reflect institutional expertise and may not be generalized to all practice settings. Although the database was prospectively maintained, the analysis is retrospective and subject to inherent selection bias. Additionally, while follow-up is relatively long (7.5 years), continued surveillance is necessary to assess for late recurrences and deaths. Patient-reported outcomes and objective cosmetic scoring were not evaluated and represent an important area for future study. We do not have accurate data regarding adjuvant chemotherapy and hormonal therapy for all patients because it was not uniformly documented in our records by the patients’ medical oncologists. We do not have specific data on multifocality/multicentricity on all patients, nor did we collect imaging-specific data. This limits our ability to report whether local recurrence was higher in patients with multifocal/multicentric disease than in those with a large primary tumor. Data were not collected on ethnicity. All patients were women.

## Conclusion

Extreme oncoplastic breast-conserving surgery allows for successful resection of large, multifocal/multicentric tumors with oncologic outcomes comparable to those of standard oncoplastic approaches. It requires careful preoperative planning and multidisciplinary consultation among breast radiology, breast surgical oncology, plastic surgery, radiation oncology, and medical oncology. In this paper, we report the largest series of extreme oncoplastic cases to date. Although the tumor burden was significantly greater, the local control, distant recurrence, and survival outcomes were comparable to those of standard cases, supporting the use of extreme oncoplastic techniques as an alternative to mastectomy in appropriately selected patients. Mastectomy should not be the default recommendation for multifocal/multicentric tumors >50 mm. Extreme oncoplasty should be offered as a definitive treatment option, in carefully selected cases, because it optimizes postoperative quality of life, eliminates the physical and psychological morbidities associated with mastectomy and prosthetic irradiation, and, most importantly, preserves the breast.
